# Recrystallization of Cellulose, Chitin and Starch in Their Individual and Native Forms

**DOI:** 10.3390/polym16070980

**Published:** 2024-04-03

**Authors:** Ekaterina Podgorbunskikh, Timofei Kuskov, Vladimir Bukhtoyarov, Oleg Lomovsky, Aleksey Bychkov

**Affiliations:** Laboratory of Mechanochemistry, Institute of Solid State Chemistry and Mechanochemistry SB RAS, 18 Kutateladze Str., 630090 Novosibirsk, Russia; t.kuskov@g.nsu.ru (T.K.); buh@solid.nsc.ru (V.B.); lomov@solid.nsc.ru (O.L.)

**Keywords:** mechanical pretreatment, disordering, crystallinity, crystal structure, cellulose, starch, chitin, lignocellulose, plant biomass

## Abstract

Semi-crystalline natural polymers are involved in many technological processes. Biopolymers having identical chemical compositions can differ in reactivity in heterogeneous transformations depending on their crystal structure (polymorphic modification). This paper compares the crystal structure recrystallization processes occurring in natural polysaccharides (cellulose, chitin, and starch) in the individual form and as a component of native biomass. Aqueous treatment of pre-amorphized semi-crystalline biopolymers was shown to result in swelling, thus alleviating the kinetic restrictions imposed on the restoration of crystalline regions and phase transition to the thermodynamically more stable polymorphic modification. During recrystallization, cellulose I in the individual form and within plant-based biomass undergoes a transition to the more stable cellulose II. A similar situation was demonstrated for α- and β-chitin, which recrystallize only into the α-polymorphic modification in the case of both individual polymers and native materials. Recrystallization of A-, B-, and C-type starch, both in the individual form and within plant-based flour, during aqueous treatment, results in a phase transition, predominantly to the B-type starch. The recrystallization process depends on the temperature of aqueous treatment; longer treatment duration has almost no effect on the recrystallization degree of polymers, both in the individual form and within native materials.

## 1. Introduction

Mechanical treatment associated with comminution, particle deformation, and accumulation of defects in the crystal lattice is an efficient method for changing the physicochemical properties of semi-crystalline polymers in the individual and native forms [1,2,3]. Thus, mechanochemical methods have shown good results for the modification and processing of lignocellulose materials [4,5,6], fabrication of nanosized and nano-structured cellulose particles, starch, and chitosan [7,8,9], amorphization and deacetylation of chitin [3,10,11,12], and other mechanochemical reactions (oxidation and etherification) [13,14].

Amorphization (AM) of the crystalline regions of biopolymers (most typically, cellulose, starch, chitin, chitosan, and β-glucan) is largely responsible for the enhanced reactivity of mechanically pretreated materials. Thus, the reduction in the crystallinity degree of cellulose is known to increase the rate of acidic and enzymatic hydrolysis [15,16], while reduced crystallinity of starch decreases its thermal stability [17,18]. The consequences of crystal structure disordering in the solid phase are relatively stable since the mobility of polysaccharide chains is limited. However, changes in the environment (e.g., during soaking, swelling, dissolution, or the subsequent heterogeneous reactions in the solid–liquid systems) increase the free volume and the degree of freedom of a molecular chain. Under these conditions, the interaction between hydroxyl groups (often attributed to the formation of hydrogen bonds) causes polysaccharide recrystallization and respective reduction of reactivity; polymorphic modification is changed in some cases. The current understanding of the self-assembly of polymer chains (cellulose, chitin, and chitosan with β-(1→4)-glycosidic linkage) indicates the dominant role of London dispersion and electrostatic interactions over hydrogen interaction forces in both intra- and inter-chain interactions [19]. Thus, it is shown that the degradation and dissolution processes are due more to London dispersion interactions than the rearrangements of hydrogen bonds.

Cellulose is the best-studied semi-crystalline polysaccharide, but the recrystallization process for cellulose has been investigated insufficiently [20]. The most abundant polymorphic modifications of cellulose are the natural cellulose I and cellulose II [20,21]. In 1941, Hess et al. were the first to demonstrate that amorphized cellulose recrystallizes to the so-called hydrated cellulose [22]. After that, the recrystallization of pure cellulose was studied rather thoroughly [23,24,25], and this process is used in such fields as the manufacturing of fibers for the textile industry (the Lyocell process) [26,27], regenerated cellulose fibers [28], or 3D printing in the food and pharmaceutical industries [29,30]. During recrystallization, cellulose undergoes a phase transition and becomes less reactive in heterogeneous transformations, especially upon hydrolysis [31]. However, the recrystallization of native cellulose-based materials remains poorly studied, thus retarding the progress in biotechnological conversion of lignocellulose biomass to low-molecular-weight carbohydrates.

Chitin naturally occurs as three polymorphic modifications: α-, β-, and γ-chitin. α-chitin is the most abundant chitin modification; it is contained in insect and crustacean exoskeletons. β-chitin is contained in the squid pen, the body of tube worms, and even some rarer sources. γ-chitin is the least common and least-studied polymorphic form of chitin, which can be isolated from fungi in minute quantities [32,33].

The term “chitin recrystallization” most typically means a phase transition of β-chitin to α-chitin in aqueous alkaline or acidic solutions (by analogy to cellulose mercerization). This can partially be attributed to the fact that intermolecular interactions in β-chitin are weaker than those in α-chitin. β-chitin is more accessible for the penetration of small polar molecules (e.g., water molecules) between the chains, thus contributing to swelling and, under certain conditions, deacetylation [34,35]. The subsequent recrystallization of β-chitin gives rise to the more energetically stable polymorphic form, α-chitin [36].

Starch can crystallize in three polymorphic modifications: A-type, B-type, and C-type [37,38,39]. Hydrothermal treatment at temperatures above 70–90 °C is known to cause starch gelatinization accompanied by the weakening molecular interactions, swelling, and melting of crystallites and double helices. The reverse process when gelatinized starch molecules are assembled into an ordered structure during drying or cooling can also take place [40]. The 40–70-monomer-long amylose double helices recrystallize relatively quickly (within the first 10–48 h). Amylopectin recrystallizes much slower (30–40 days), and shorter helices are formed. It has been demonstrated for individual starch samples that upon hydrothermal treatment, B- and C-type starch can be transformed into A-type starch. Meanwhile, the transformation of A-type starch into other crystalline forms takes place only after the original structure is completely disordered (amorphized) [37,39,40].

The recrystallization of starch is practically used in the food and biomedical industries. Slowly digestible starch (SDS) and resistant starch (RS), which are produced by the hydrolysis of rapidly digestible starch (RDS) or recrystallization, affect the glycemic and insulinemic responses and reduce the risk of developing diabetes while supporting the cardiovascular system, well-being, performance, etc. [41,42]. Meanwhile, the recrystallization of starch contained in crop flour has been poorly studied; the procedures of the hydrothermal treatment of starch-based feedstocks commonly used in the food industry and biotechnology are performed scholastically, without appealing to knowledge about the polymer structure.

Phase transitions are the least studied factor influencing the reactivity of semi-crystalline carbohydrates. It is generally believed that the specific surface area is responsible for the reaction rate in heterogeneous transformations, and the degree of disorder of the crystalline structure is responsible for the reaction yield [16,43]. The notion that pre-amorphized polymers possess the ability to restore structure (and occasionally alter polymorphic modification) is typically regarded separately from the study of reactivity, as an independent process. The notion that pre-amorphized polymers possess the ability to restore structure and occasionally alter polymorphic modification is typically regarded separately from the study of reactivity, as an autonomous process. A few instances clearly demonstrate the erroneousness of this approach, both from the standpoint of chemistry (in the event that two distinct polymorphs exhibit identical crystallinity, their reactivity levels may differ) and from the standpoint of technology (the expenditure of energy and money on pre-amorphization is futile as it may result in the polymer’s transition to a less reactive polymorphic modification upon contact with water). On the basis of acid hydrolysis of amorphized cellulose I, it was demonstrated that in an aqueous environment, there is a rapid recrystallization and phase transition into a mixture of cellulose I and II. Recrystallized cellulose has lower reactivity but a higher rate of cellulose recrystallization than the hydrolysis reaction [44].

A similar effect was observed with chitin. It is known that the reactivity of amorphized chitin is higher [3], and there is a difference between the reactivity of α- and β-chitin. In this regard, it is necessary to consider the influence of chitin polymorphism on the deacetylation kinetics.

In the case of starch, the process of amorphization and recrystallization facilitates the control of biotechnological processes aimed at obtaining digestible starch and resistant starch, which have opposing functions. The variation in the chemical composition (source) of starch under the same heterogeneous conditions will lead to different rearrangements of the structure.

X-ray powder diffraction (XRD) with subsequent analysis of patterns is most often used to study the crystal structure, amorphization, and recrystallization processes of semi-crystalline polysaccharides (cellulose, chitin, and starch) because the equipment is readily available. XRD techniques allow the identification of the crystalline phase, and methods developed to evaluate the crystallinity index of polysaccharides allow for comparisons, both within a single experiment and by using data for different polymer sources [45]. In this work, we applied to the corresponding polymers the most frequently used methods of crystallinity index estimation, which, by taking into account the peculiarities and limitations of each method within one experimental set of samples, allow for a rapid assessment of the processes taking place.

The semi-crystalline polysaccharides (cellulose, chitin, and starch) have close structures (β-1-4-linked in cellulose and chitin and α-1-4- and α-1-6-linked in starch), which account for the difference in reactivity between their polymorphic modifications. Chitosan is an artificial derivative of chitin and, therefore, was not considered in this work. Even for the relatively well-studied recrystallization of individual polysaccharides, the experimental results are poorly systematized since they have been obtained, recorded, and mathematically processed under different conditions. The recrystallization of native polysaccharide-based objects remains virtually uninvestigated.

Hence, this study aimed to perform methodologically homogeneous comparisons of the recrystallization of mechanically amorphized cellulose, chitin, and starch in their individual form and within native biomass. The investigation of the contribution of partial crystallinity recovery, as well as phase transitions, to heterogeneous processes of biopolymer processing can aid in the further enhancement of structure-reactivity relationships, as well as the development of processes that exclude the recrystallization of cellulose, production of chitosan, and resistant starch. The presented material will possess significant value in the development of “starch—chitosan” or “starch—cellulose” polymer composites, and, particularly, in the creation of multicomponent biodegradable plastics.

## 2. Materials and Methods

### 2.1. Materials

The following materials were used in this study: cellulose (CAS C6288, Sigma-Aldrich, Moscow, Russia), pine sawdust (*Pinus sylvestris*) (Novosibirsk region, Russia, harvested in July 2022), chitin from shrimp shells (CAS 1398-61-4, Sigma-Aldrich), shell of the red king crab (*Paralithodes camtschaticus*) (wild-caught on the eastern coast of the Kamchatka Peninsula in July 2022), pens of squid (*Loligo vulgarus*) (wild-caught on the eastern coast of the Kamchatka Peninsula in July 2022), corn starch (State Standard GOST 32159-2013, top grade, Garnec OJSC, Vladimir, Russia), potato starch (State Standard GOST R 53876-2010, top grade, Garnec OJSC, Vladimir, Russia), cassava starch (State Standard GOST 32159-2013, top grade, Garnec OJSC, Vladimir, Russia), corn flour (technical specification TU 10.61.20-001-32916290, OJSC MAKFA, Chelyabinsk, Russia), potato tubers (the Idaho high-starch variety, Novosibirsk region, 2022 crop), cassava flour (technical specification TU 10.61.23-010-42764013-2020, OJSC Fabrika Zdorovya, Moscow, Russia), NaOH (KHIMMED, Moscow, Russia), HCl (Khimprom, Moscow, Russia), and acetone (pure for analysis grade, KHIMMED, Moscow, Russia).

### 2.2. Extraction of β-Chitin

According to the procedure described in ref. [46], squid pens were washed with distilled water and comminuted into 5–10-mm fragments using a laboratory knife grinder; then, they were demineralized with 0.55 M HCl solution (hydromodulus of 1:10) at room temperature for 2 h. Deproteinization using 1 M NaOH solution was conducted at 80 °C for 10 h (hydromodulus of 1:20). The reaction mass was mixed on a magnetic stirrer at a rate of 600 rpm at each stage. After the reaction, the insoluble precipitate was filtered off and dried in a vacuum drying oven at 55 °C for 24 h.

### 2.3. Preparation of Potato Flour

Potatoes, preliminarily washed and peeled, were comminuted in a laboratory knife grinder to the size of 2–5 mm and frozen at –20 °C. The frozen ground potatoes were freeze-dried for 24 h on an Iney-4 laboratory freeze dryer (Institute for Biological Instrumentation, Pushchino, Russia).

### 2.4. Mechanical Treatment

Mechanical treatment of all samples was carried out in an AGO-2 water-cooled laboratory planetary ball mill (Institute of Solid State Chemistry and Mechanochemistry SB RAS, Novosibirsk, Russia) (grinding media: steel balls of 5 mm in diameter, weighing 200 g; grinding body acceleration, 200 m/s^2^; and nominal motor power, 1.1 kW). The weight of the treated samples was 10 g; the treatment duration was 600 s for the starch-based materials and 2700 s for cellulose- and chitin-based materials.

### 2.5. Recrystallization

To study polysaccharide recrystallization, pre-amorphized samples were treated with water at a hydromodulus of 1:10 at 25 °C and 95 °C for 1, 4, 8, 12, and 24 h. The fluid was then removed by centrifuging; the precipitate was washed with acetone to remove residual water and dried at 70 °C until the weight stopped changing.

The recrystallization degree was determined using Equation (1):

RD = 100 − 100% × (CrI_0_ − CrI_t_)/CrI_0_, (1)

where RD is the recrystallization degree, CrI_0_ is the initial crystallinity index, and CrI_t_ is the crystallinity index after recrystallization at instant *t*.

### 2.6. Scanning Electron Microscopy (SEM)

The particle morphologies of the initial and amorphized samples were characterized by scanning electron microscopy (SEM) on a TM-1000 microscope (Hitachi, Tokyo, Japan) at an accelerating voltage of 15 kV. A gold coating was deposited onto the sample surface using a JFC-1600 magnetron sputter coater (Jeol, Tokyo, Japan) (deposition time, 40 s; ion current, 30 mA; and coating thickness, 10 nm).

### 2.7. Particle Size

The particle size of the initial and amorphized samples was measured on a CAMSIZER X2 optical analyzer (Retsch GmbH, Haan, Germany) equipped (detection threshold, 0.8–8000 µm) with a compressed air dispersion module X-Jet (under a pressure of 60 kPa). The average particle size was determined by image (1000 images) analysis in compliance with ISO 13322-2:2006 [47]. Furthermore, we determined a sphericity parameter b/l, which is the ratio between the minimal and the maximal inscribed chords, according to ISO 13322-2:2021 [48].

### 2.8. Specific Surface Area (SSA)

The specific surface area (SSA) of the samples was (nearly 1 g) measured according to the thermal desorption of nitrogen at 77 K on a Sorptometer M instrument (Catakon, Novosibirsk, Russia). SSA was determined by the Brunauer–Emmett–Teller (the BET method) multipoint method by using at least five data points [49].

### 2.9. X-ray Diffraction (XRD) Analysis

The crystal structures of the initial, amorphized, and recrystallized samples were characterized by X-ray diffraction (XRD) analysis on a D8 Advance diffractometer (Bruker, Germany) with monochromatic CuKα radiation in the Bragg–Brentano reflection geometry. The step size was 0.0195°. The analysis was performed in a broad range of 2θ angles (5–60°) at a voltage of 40 kV and current of 40 mA. The X-ray wavelength was 1.5406 Å.

The crystallinity index (CrI) of cellulose-based materials was determined using the peak height method (Segal method) [45,50], which relates the (200) reflection intensity (crystallinity peak) to the intensity of the minimum between the (110) and (200) reflections (noncrystalline, I_AM_) after subtracting the background signal (Equation (2)):

CrI = 100% × (I_200_ − I_AM_)/I_200_. (2)

The crystallinity index of starch-based materials was calculated using the earlier proposed modification of Nara and Komiya’s method [51,52]. A smoothed curve connecting the peak baselines was superimposed onto the recorded XRD pattern. The area above the smoothed curve corresponded to the crystalline portion of starch, while the area under the curve corresponded to the amorphous one. The crystallinity index was calculated as the ratio between the area of the crystalline phase and the total area under the XRD curve using Equation (3):

CrI = 100% × S_(cr.phase)_/S_(total)_, (3)

where S_(cr.phase)_ is the area of the crystalline phase and S_(total)_ is the total area under the XRD curve.

The crystallinity index of chitin-based materials was determined using the method proposed by Focher [53], which was based on the ratio between the intensities of the most crystalline reflection with minimum intensity at 2θ = 16°, which conditionally describes the diffuse halo maximum (I_AM_) (Equations (4) and (5)). For α-chitin, the intensity of (110) reflection was measured at 19–20° (Equation (4)); for β-chitin, the intensity of (1-10) reflection was measured at ~20° (Equation (5)).

CrI = 100% × (I_110_ − I_AM_)/I_110_, (4)

CrI = 100% × (I_1-10_ − I_AM_)/I_1-10_. (5)

### 2.10. Statistical Analysis

In each assay, repeats were performed at least 3 times on different representative samples. Data were expressed as mean ± standard deviation (SD).

## 3. Results and Discussion

### 3.1. Mechanical Treatment

The primary objective of this study was to obtain amorphized polysaccharide samples differing in composition, structure, and, therefore, structure resistance to mechanical treatment. It was demonstrated earlier that to be completely amorphized, linear polysaccharides need to be subjected to a longer mechanical treatment than the branched ones. The time of amorphization of cellulose and chitosan in a planetary ball mill is longer than 30 min, while complete amorphization of starch under identical conditions takes 10 min [38,52,54]. Therefore, the following conditions for obtaining completely amorphous polysaccharides in the planetary ball mill were selected in this study: 45 min for cellulose- and chitin-based materials and 10 min for starch-based materials.

**Figure 1:**
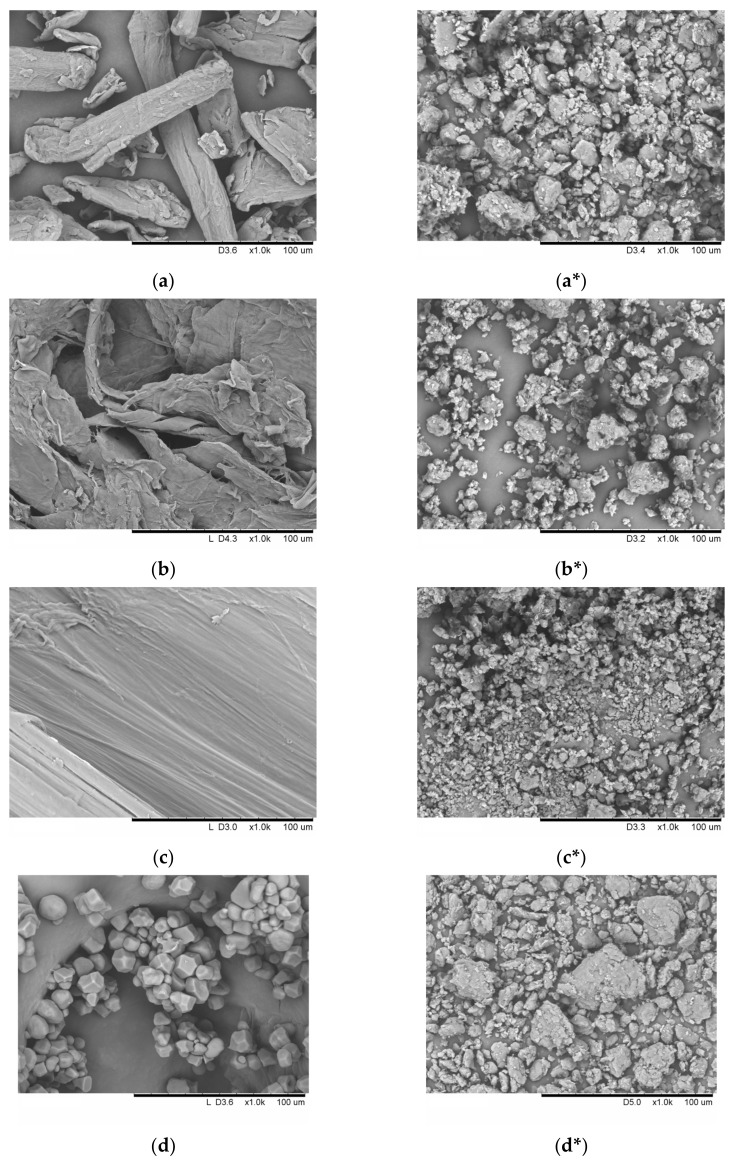

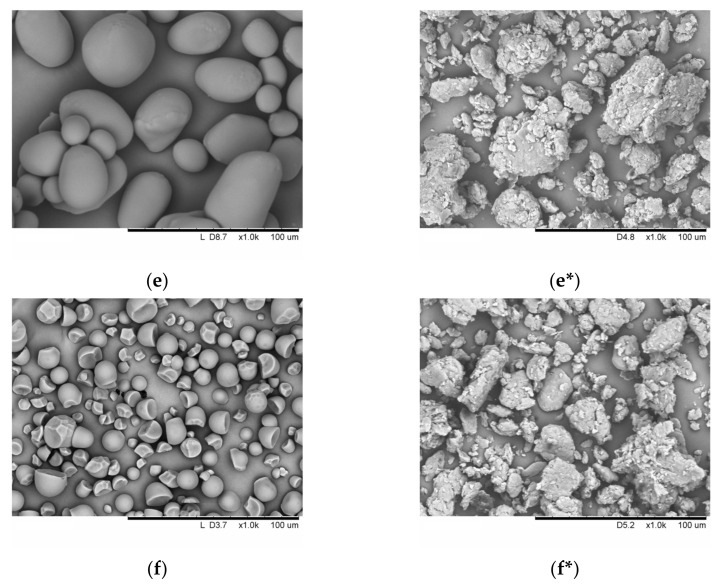
The SEM morphology of the initial and amorphized individual polymers: (**a**,a*) cellulose; (**b**,b*) α-chitin; (**c**,c*) β-chitin; (**d**,d*) A-type starch; (**e**,e*) B-type starch; and (**f**,f*) C-type starch. *—amorphized samples.

The SEM micrographs of the individual polymers (cellulose I, α-chitin, β-chitin, and starch) before and after mechanical treatment are shown in Figure 1. Cellulose and chitin are linear polymers, which is evident from the morphology of the particles. The morphology of these polymers is characterized by a regular fibrous structure, and there are few differences between them (Figure 1a–c). In turn, Figure 1d–f shows the SEM micrographs of granules of corn, potato, and cassava starches in the individual form. Corn starch granules (A-type) are irregular polygons characterized by concave edges, as depicted in Figure 1d. Potato starch granules (B-type) are predominantly spherical or elliptical in shape (Figure 1e). Tapioca starch granules (C-type) are spherical or irregularly shaped, exhibiting a combination of A- and B-type morphology (Figure 1f).

After undergoing mechanical treatment for 2700 s for cellulose, α-chitin, and β-chitin and mechanical treatment for 600 s for starches, it has been observed that the particles of all individual polymers under study undergo modification due to impact-shear action, resulting in a decrease in particle size and aggregation (Figure 1a*–f*). The particle sizes, sphericity parameters (b/l), and specific surface area (SSA) of the studied individual polymers before and after amorphization are presented in Table 1. After mechanical treatment, particles of linear polymers (cellulose, α-chitin, and β-chitin) acquire a more spherical shape (b/l > 0.7) and become disordered and particle sizes decrease. The opposite situation is observed for starch samples; the granules are strongly deformed under mechanical force, and they flatten and form stable aggregates. The sphericity parameters decrease (b/l < 0.8), and the particles take the shape of a rod. Specific surface area increases for all polymers, and mechanical treatment can increase SSA up to three times.

### 3.2. Recrystallization

The recrystallization of pre-amorphized samples was carried out at room (25 °C) and elevated (95 °C) temperatures for 1–24 h. Recrystallization was stopped by washing the samples with acetone, which resulted in water removal and prevented the uncontrolled crystal structure rearrangement when the samples were dried. Samples in the individual (purified) and native forms (cellulose as a component of wood, chitin within shells and skeletons of marine fauna species, and starch as agricultural crop flour) were obtained under these conditions and characterized.

**Figure 2:**
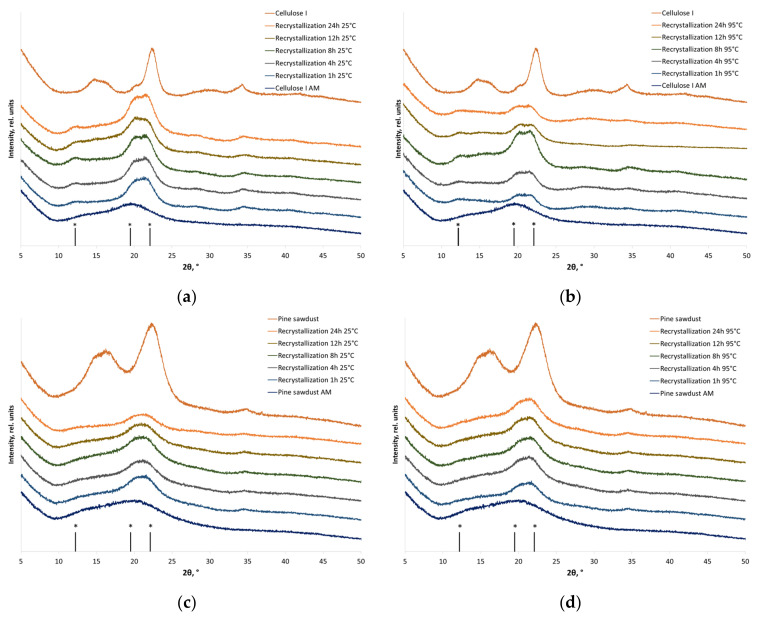
The XRD patterns of the initial, amorphized, and recrystallized cellulose-based materials: (**a**) individual cellulose (recrystallization at 25 °C); (**b**) individual cellulose (recrystallization at 95 °C); (**c**) cellulose in pine sawdust (recrystallization at 25 °C); and (**d**) cellulose in pine sawdust (recrystallization at 95 °C). * Positions of cellulose II reflections.

Figure 2 shows the XRD patterns of individual cellulose (cellulose I) and plant biomass (pine sawdust). One can see that cellulose in the plant-based objects is represented by cellulose I [56], with the major crystal plane reflections at 2θ = 14.8°, 16.7° and 23.0° corresponding to Miller indices ($\bar{1}10$), (110), and (200) (adopted for cellulose Iβ). For comparison, cellulose II is characterized by positions of the ($\bar{1}10$), (110), and (020) reflections at 2θ = 12.2°, 19.5°, and 22.1° [56]. Similar changes in powder XRD patterns are typical of all the cellulose-based materials subjected to mechanical treatment: the crystallographic peaks of cellulose broaden substantially, their intensity decreases, and an amorphous halo with the characteristic maximum at 19–20° is formed (Figure 2).

The recrystallization of individual cellulose samples subjected to treatment with water at 25 and 95 °C contributes to the recovery of crystalline regions, which manifests itself as increased intensity of XRD peaks and crystallinity indices (Table 2). After recrystallization, amorphous cellulose I is a mixture of polymorphic modifications of cellulose I and II (Figure 2a,b), which was demonstrated earlier by Liang et al. [57] under similar conditions. The depth of cellulose structure recovery depends on the treatment temperature. The crystallinity index increases from 13 to 64% at 25 °C and to 71% at 95 °C, corresponding to the recrystallization degrees of 72% and 78%, respectively.

Amorphized cellulose I within a plant-based material is also converted to a mixture of cellulose I and II upon recrystallization (Figure 2c,d). The temperature of aqueous treatment does not considerably affect the rearrangement of parallel polymer chains in cellulose I into antiparallel cellulose II.

Recrystallization proceeds relatively quickly both for individual cellulose and the plant-based samples (much faster than it was demonstrated in ref. [32]); the duration of aqueous treatment has no significant effect on the crystallization index (Table 2). However, like in the case of individual cellulose, the recrystallization degree for pine sawdust is temperature-dependent (65% at 25 °C and 71% at 95 °C).

Another linear polysaccharide characterized by polymorphism is chitin; its main polymorphic forms are known as α-chitin and β-chitin. The most common polymorphic form, α-chitin, is characterized by antiparallel packing of polymorphic chains, similar to that in cellulose II, which explains the high crystallinity and structural stability. β-chitin chains are packed in a parallel manner, like in cellulose I, which makes β-chitin more reactive and less abundant in nature.

**Figure 3:**
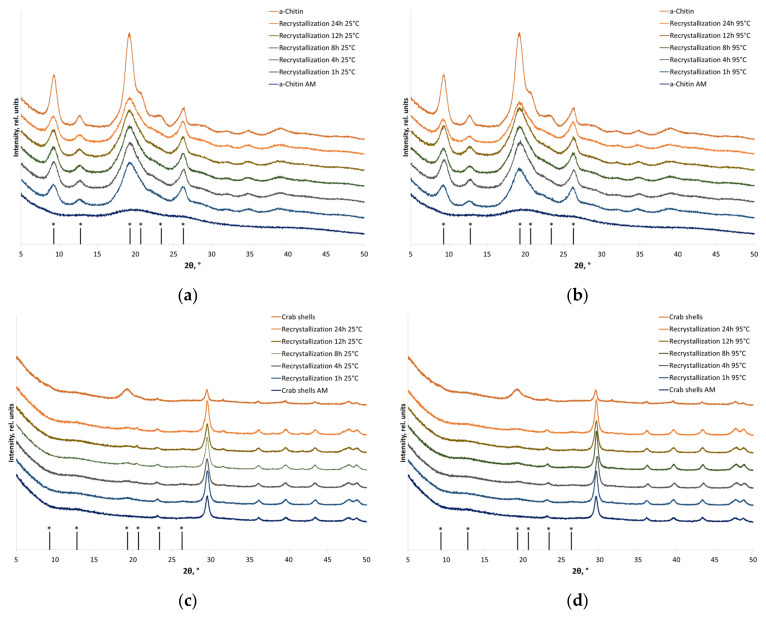
The XRD patterns of the initial, amorphized, and recrystallized chitin-based materials: (**a**) individual α-chitin (recrystallization at 25 °C); (**b**) individual α-chitin (recrystallization at 95 °C); (**c**) α-chitin within crab shells (recrystallization at 25 °C); and (**d**) α-chitin within crab shells (recrystallization at 95 °C). * Positions of α-chitin reflections.

In the XRD patterns of the initial α-chitin, one can clearly see reflections at 2θ = 9.3°, 12.8°, 19.3°, 20.7°, 23.4°, and 26.3°, which correspond to reflections (020), (021), (110), (120), (130), and (013) of α-chitin crystals [41]. After the aqueous treatment of amorphized α-chitin, the diffraction image was restored (Figure 3), without changes in the polymorphic modification, for α-chitin, both in the individual form and within natural feedstock. The recrystallization degree of individual α-chitin was 82% of the initial crystallinity (Table 3). The recrystallization degree of α-chitin within the crab shell was ~30% (the accurate value could not be measured since the reflections of α-chitin within the natural feedstock are strongly overlapped by reflections corresponding to the mineral part of the shell).

The XRD patterns of β-chitin contain two major and two minor peaks at 2θ = 8.5°, 12.6°, 19.5°, 20.7°, and 27.0°, which correspond to reflections (010), (011), ($1\bar{1}0$), (021), and (013) [41]. Well-defined peaks at 2θ = 9.3°, 12.8°, 19.3°, and 26.7°, corresponding to the reflections of α-chitin (020), (021), (110), and (130), respectively, appear in the XRD patterns after recrystallization. The crystallinity index of individual β-chitin after recrystallization to α-chitin was restored to 78% of the initial value (regardless of temperature), while the crystallinity of chitin as a component of squid pens at 25 °C and 95 °C was restored to 63% and 73%, respectively (Table 3).

**Figure 4:**
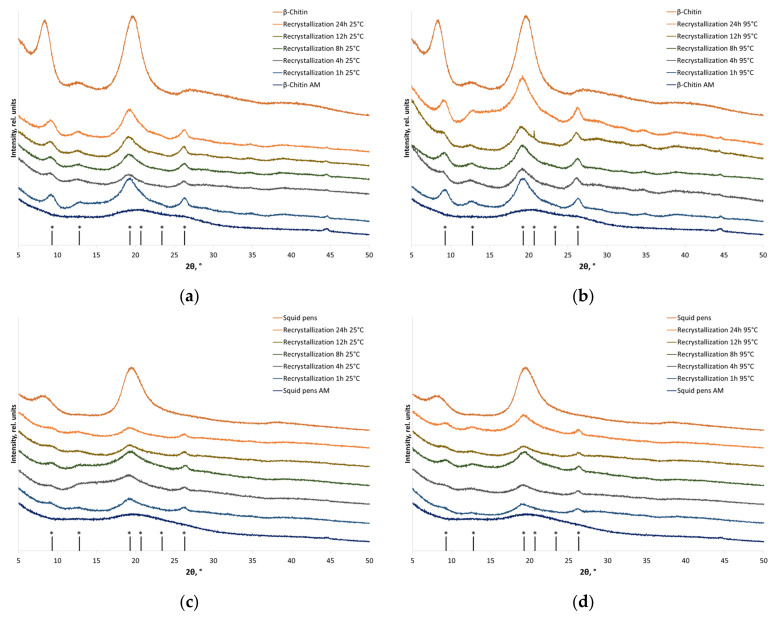
X-ray diffraction patterns of the initial, amorphized, and recrystallized chitin-based materials: (**a**) individual β-chitin (recrystallized at 25 °C); (**b**) individual β-chitin (recrystallized at 95 °C); (**c**) β-chitin in squid pens (recrystallized at 25 °C); and (**d**) β-chitin in squid pens (recrystallized at 95 °C). * Positions of α-chitin reflections.

The recrystallization of pre-amorphized β-chitin to α-chitin (Figure 4) is consistent with the phase transitions of highly crystalline chitin from squid that, earlier, were shown to occur in aqueous alkaline solutions [34,36]. Huang et al. [36] attributed the phase transition of β-chitin to α-chitin to the ability of β-chitin to form a hierarchical corrugated board structure of chains, contributing to the self-assembly into a more energetically stable form (α-chitin) after the interaction with solvent molecules.

Molecular dynamics simulation demonstrated differences in the interaction of α- and β-chitin with water molecules. Hydrogen bonds in α-chitin stabilize the interactions between parallel chains, thus forming a barrier that impedes the free motion of water molecules inside the layers. In contrast, it has been demonstrated for β-chitin that water molecules possess the ability to move, thereby augmenting the mobility of chitin chains and disrupting their linear organization. It causes disruption of the β-chitin structure and its transition to the α-chitin structure [35].

**Figure 5:**
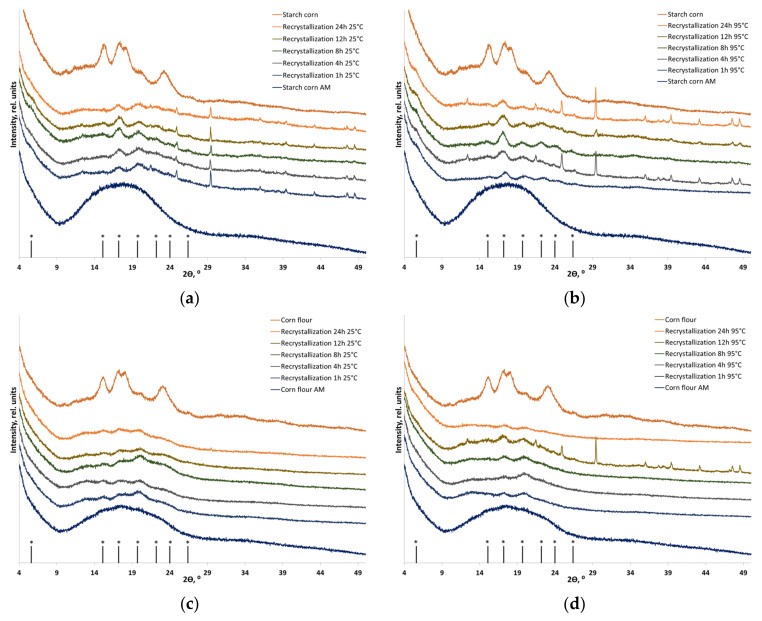
The XRD patterns of the initial, amorphized, and recrystallized starch-based materials: (**a**) A-type starch (individual) recrystallized at 25 °C; (**b**) A-type starch (individual) recrystallized at 95 °C; (**c**) A-type starch (within corn flour) recrystallized at 25 °C; and (**d**) A-type starch (within corn flour) recrystallized at 95 °C. * Positions of B-type starch reflections.

**Figure 6:**
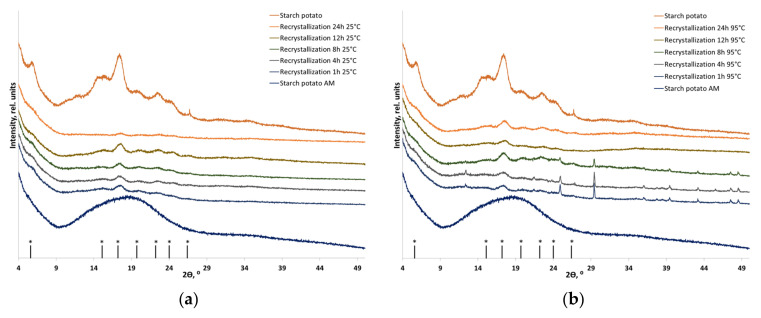

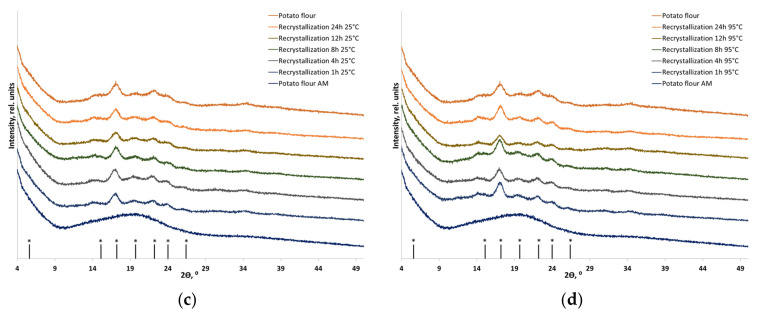
The XRD patterns of the initial, amorphized, and recrystallized starch-based materials: (**a**) B-type starch (individual) recrystallized at 25 °C; (**b**) B-type starch (individual) recrystallized at 95 °C; (**c**) B-type starch (within potato flour) recrystallized at 25 °C; and (**d**) B-type starch (within potato flour) recrystallized at 95 °C. * Positions of B-type starch reflections.

**Figure 7:**
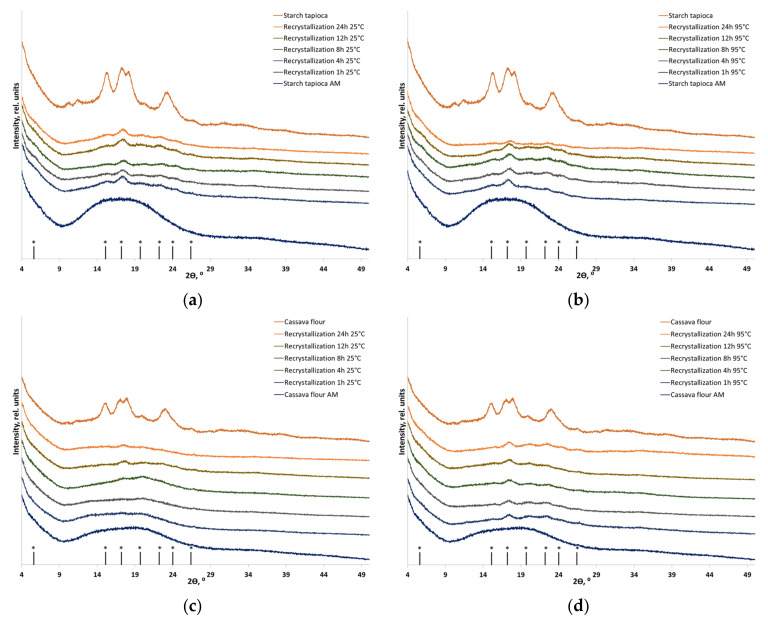
The XRD patterns of the initial, amorphized, and recrystallized starch-based materials: (**a**) B-type starch (individual) recrystallized at 25 °C, (**b**) C-type starch (individual) recrystallized at 95 °C; (**c**) C-type starch (within cassava flour) recrystallized at 25 °C; and (**d**) C-type starch (within cassava flour) recrystallized at 95 °C. * Positions of B-type starch reflections.

Figure 5, Figure 6 and Figure 7 show the XRD patterns of corn, potato, and cassava starches in their individual forms and as components of plant-based biomass. The diffraction patterns of corn, potato, and cassava starches correspond to the A-, B-, and C-type polymorphic modifications, respectively [38]. The shape of the diffraction image is determined by amylopectin, which can crystallize in three main polymorphic modifications (amylose in native starch is usually X-ray amorphous). The XRD pattern of corn starch and corn flour contains diffraction peaks at 2θ = 15.0°, 17.0°, 17.9°, and 22.9°, which correspond to the A-type crystalline structure (Figure 5). The XRD patterns of potato starch and potato flour contain diffraction peaks at 2θ 5.6°, 15.1°, 17.2°, 19.7°, 22.2°, 24.0°, and 26.4°, corresponding to the B-type crystalline structure (Figure 5). The C-type crystalline structure consists of A- and B-type crystallites; the XRD patterns of cassava starch and cassava flour contain diffraction peaks at 2θ = 15.2°, 17.12°, 18.19°, and 22.8° (Figure 7). After mechanical treatment, an amorphous halo appears due to the disordering of the starch structure. Although differing in shape, the XRD patterns of amorphized corn, potato, and cassava flour are similar to that of amorphized lignocellulosic biomass because they have a more versatile composition and contain a certain amount of non-starch polymers. Table 4 summarizes the crystallinity indices of the initial and recrystallized samples.

One can see that the crystallinity of individual A-type, B-type, and C-type starches after amorphization followed by recrystallization is partially restored, and transition, predominantly to the B-type starch, takes place in all the cases. Quantitative analysis and dependable evidence of the absence of A-type starch in the recrystallization product are currently unfeasible and necessitate precise deciphering of the crystal structure, followed by the Rietveld method [58]. Hence, the recrystallization of amorphized corn starch (A-type) resulted in the formation of B-type starch. Furthermore, recrystallization at a temperature close to the gel point (95 °C) increased the recrystallization degree to 44% vs. 35% after treatment at 25 °C. Potato starch (B-type) does not undergo a phase transition, but its crystallinity is restored to 48% at 25 °C and to 54% at 95 °C. The diffraction pattern of individual cassava starch also indicates that a phase transition between C-type and B-type starches takes place (Figure 7a,b); crystallinity is restored to the level of 23% of the initial one.

During recrystallization, amorphized starch within flour also undergoes a phase transition to the B-type (the detection of the presence and quantification of A-type starch in the case of flour is also not possible), whose recrystallization depends on temperature and aqueous treatment. The recrystallization degrees of starch within corn flour (A-type) at 25 °C and 95 °C were 24% and 29% of the initial one, respectively. The crystallinity of starch within potato flour (B-type) was restored to 54% and 60% at 25 °C and 95 °C, respectively, without a phase transition. Starch within cassava flour (C-type), which is considered to be a mixture of A-type and B-type starches, is characterized by the lowest degree of crystallinity recovery: after the phase transition to B-type starch, it was 8% at room temperature and 22% at 95 °C.

Considering the findings above, it can be concluded that water treatment of pre-amorphized biopolymers leads to a partial restoration of the ordering of the crystal structure with a phase transition to a more stable polymorphic modification (Table 5). The degree of recrystallization is contingent upon the temperature of the treatment but not on its duration. The behavior of the phase transition after deep amorphization of the substance does not depend on the supramolecular composition of the material.

## 4. Conclusions

Treatment of pre-amorphized semi-crystalline carbohydrate biopolymers with water is accompanied by swelling, which is related to the solvation of polymer chains by water molecules. Therefore, kinetic limitations for the recovery of crystalline regions and, in some cases, for transition to the thermodynamically more stable polymorphic modification, are alleviated.

Amorphized cellulose I in the individual form and as a component of plant-based biomass recrystallizes to the more stable cellulose II. A similar situation was observed for α- and β-chitin, which recrystallize exclusively to the α-polymorphic modification in the case of both individual polymers and native materials. The recrystallization of A-, B-, and C-type starch, both in the individual form and as a component of plant-based flour, causes the phase transition, predominantly to B-type starch, under the described aqueous treatment conditions.

The degree of crystal structure restoration increased with treatment temperature; however, the duration of aqueous treatment had almost no effect on the recrystallization degree of polymers, both in the individual form and as a component of native materials.

The demonstrated patterns should be taken into account not only when elaborating the mechanochemical technologies for processing cellulose-, chitin-, and starch-based feedstocks but also for manufacturing artificial copolymeric materials, which are currently popular among researchers.

## Figures and Tables

**Table 1:** Physicochemical parameters of the initial and amorphized individual polymers: cellulose, α-chitin, β-chitin, and A-, B- and C-type starch.

Sample	Average Particle Size, μm	Sphericity, b/l	SSA ^1^, m^2^/g	Ref.
Individual cellulose				
Before amorphization	39.0 ± 0.3	0.562 ± 0.001	1.6 ± 0.2	present work
After amorphization	29.4 ± 0.1	0.768 ± 0.003	2.1 ± 0.2	present work
Individual α-chitin				
Before amorphization	120.3 ± 0.4	0.606 ± 0.001	6.3 ± 0.3	present work
After amorphization	19.8 ± 0.1	0.779 ± 0.002	2.7 ± 0.2	present work
Individual β-chitin				
Before amorphization	52.5 ± 0.3	0.599 ± 0.001	6.6 ± 0.1	present work
After amorphization	19.3 ± 0.1	0.778 ± 0.002	3.1 ± 0.2	present work
Individual starch (A-type)				
Before amorphization	19.0 ± 0.1	0.798 ± 0.001	0.7 ± 0.1	[55]
After amorphization	23.8 ± 0.1	0.772 ± 0.001	1.6 ± 0.1	[55]
Individual starch (B-type)				
Before amorphization	28.4 ± 0.1	0.780 ± 0.001	0.5 ± 0.1	[55]
After amorphization	29.7 ± 0.1	0.753 ± 0.001	1.5 ± 0.1	[55]
Individual starch (C-type)				
Before amorphization	16.6 ± 0.1	0.839 ± 0.001	0.8 ± 0.1	[55]
After amorphization	27.5 ± 0.1	0.758 ± 0.001	1.5 ± 0.1	[55]

Data are presented as mean ± SD. ^1^ SSA—specific surface area.

**Table 2:** The crystallinity index of the initial, amorphized, and recrystallized cellulose-based materials.

Sample	CrI, %	
Individual cellulose		
Before amorphization	90 ± 2	
After amorphization	<<13	
Recrystallized individual cellulose		
	25 °C	95 °C
Recrystallization for 1 h	71 ± 3	75 ± 2
Recrystallization for 4 h	69 ± 3	77 ± 2
Recrystallization for 8 h	64 ± 2	67 ± 4
Recrystallization for 12 h	53 ± 4	68 ± 2
Recrystallization for 24 h	65 ± 4	67 ± 3
Cellulose in pine sawdust		
Before amorphization	60 ± 2	
After amorphization	<<13	
Recrystallized cellulose in pine sawdust		
	25 °C	95 °C
Recrystallization for 1 h	39 ± 3	43 ± 3
Recrystallization for 4 h	38 ± 4	42 ± 2
Recrystallization for 8 h	37 ± 4	43 ± 2
Recrystallization for 12 h	41 ± 2	44 ± 1
Recrystallization for 24 h	46 ± 3	43 ± 3

**Table 3:** The crystallinity indices of the initial, amorphized, and recrystallized chitin-based materials.

Sample	CrI, %	
Individual α-chitin		
Before amorphization	80 ± 1	
After amorphization	<<16	
Recrystallized individual α-chitin		
	25 °C	95 °C
Recrystallization for 1 h	65 ± 2	65 ± 4
Recrystallization for 4 h	64 ± 2	65 ± 2
Recrystallization for 8 h	67 ± 2	67 ± 2
Recrystallization for 12 h	65 ± 3	65 ± 2
Recrystallization for 24 h	65 ± 2	65 ± 2
α-Chitin in crab shells		
Before amorphization	42 ± 4	
After amorphization	<<10	
Recrystallized α-chitin in crab shells		
	25 °C	95 °C
Recrystallization for 1 h	11 ± 4	10 ± 4
Recrystallization for 4 h	13 ± 3	10 ± 3
Recrystallization for 8 h	10 ± 2	18 ± 3
Recrystallization for 12 h	12 ± 4	19 ± 3
Recrystallization for 24 h	12 ± 4	14 ± 4
Individual β-chitin		
Before amorphization	62 ± 2	
After amorphization	<<12	
Recrystallized individual β-chitin		
	25 °C	95 °C
Recrystallization for 1 h	47 ± 4	55 ± 4
Recrystallization for 4 h	36 ± 3	40 ± 3
Recrystallization for 8 h	45 ± 2	55 ± 3
Recrystallization for 12 h	52 ± 3	50 ± 4
Recrystallization for 24 h	55 ± 4	41 ± 4
β-Chitin in squid pens		
Before amorphization	48 ± 2	
After amorphization	<<12	
Recrystallized β-chitin in squid pens		
	25 °C	95 °C
Recrystallization for 1 h	35 ± 3	40 ± 4
Recrystallization for 4 h	21 ± 4	28 ± 4
Recrystallization for 8 h	32 ± 3	37 ± 3
Recrystallization for 12 h	33 ± 2	33 ± 2
Recrystallization for 24 h	29 ± 3	39 ± 3

**Table 4:** The crystallinity index of the initial, amorphized, and recrystallized starch-based materials.

Sample	CrI, %	
Individual starch (A-type)		
Before amorphization	34 ± 2	
After amorphization	<<2	
Recrystallized individual starch (A-type)		
	25 °C	95 °C
Recrystallization for 1 h	8 ± 3	14 ± 4
Recrystallization for 4 h	9 ± 2	12 ± 4
Recrystallization for 8 h	15 ± 3	18 ± 3
Recrystallization for 12 h	15 ± 2	18 ± 2
Recrystallization for 24 h	12 ± 3	13 ± 3
A-starch in corn flour		
Before amorphization	29 ± 2	
After amorphization	<<2	
Recrystallized A-starch in corn flour		
	25 °C	95 °C
Recrystallization for 1 h	5 ± 3	8 ± 4
Recrystallization for 4 h	8 ± 3	9 ± 3
Recrystallization for 8 h	7 ± 2	9 ± 3
Recrystallization for 12 h	9 ± 3	8 ± 4
Recrystallization for 24 h	9 ± 4	9 ± 4
Individual starch (B-type)		
Before amorphization	27 ± 2	
After amorphization	<<2	
Recrystallized individual starch (B-type)		
	25 °C	95 °C
Recrystallization for 1 h	12 ± 2	14 ± 2
Recrystallization for 4 h	13 ± 2	14 ± 2
Recrystallization for 8 h	13 ± 2	15 ± 3
Recrystallization for 12 h	13 ± 3	15 ± 2
Recrystallization for 24 h	14 ± 2	15 ± 3
B-starch in potato flour		
Before amorphization	18 ± 4	
After amorphization	<<2	
Recrystallized B-starch in potato flour		
	25 °C	95 °C
Recrystallization for 1 h	10 ± 2	10 ± 3
Recrystallization for 4 h	11 ± 2	12 ± 2
Recrystallization for 8 h	8 ± 3	8 ± 2
Recrystallization for 12 h	10 ± 3	13 ± 2
Recrystallization for 24 h	10 ± 2	11 ± 3
Individual starch (C-type)		
Before amorphization	40 ± 2	
After amorphization	<<2	
Recrystallized individual starch (C-type)		
	25 °C	95 °C
Recrystallization for 1 h	8 ± 2	6 ± 3
Recrystallization for 4 h	10 ± 2	8 ± 2
Recrystallization for 8 h	10 ± 2	9 ± 2
Recrystallization for 12 h	9 ± 3	10 ± 2
Recrystallization for 24 h	10 ± 2	13 ± 2
C-starch in cassava flour		
Before amorphization	32 ± 2	
After amorphization	<<2	
Recrystallized C-starch in cassava flour		
	25 °C	95 °C
Recrystallization for 1 h	2 ± 1	4 ± 1
Recrystallization for 4 h	2 ± 1	6 ± 2
Recrystallization for 8 h	2 ± 1	9 ± 1
Recrystallization for 12 h	4 ± 2	7 ± 2
Recrystallization for 24 h	3 ± 1	8 ± 2

**Table 5:** Schematic of phase transitions after recrystallization of amorphized polymers (cellulose, chitin, and starch) in individual and native forms.

Before Amorphization and Recrystallization	More (+) and Less (−) Stable Polymorphic Modification	After Recrystallization	
		In Individual Form	In Native Form
Linear polysaccharides			
Cellulose I	−	I + II	I + II
Cellulose II	+	II	II
α-Chitin	+	α-chitin	α-chitin
β-Chitin	−	α-chitin	α-chitin
Branched polysaccharides			
Starch (A-type)	−	B-type *	B-type *
Starch (B-type)	+	B-type *	B-type *
Starch (C-type)	−	B-type *	B-type *

*—predominant polymorph.

## Data Availability

Data are contained within the article.

## References

[1] Baláž P., Achimovičová M., Baláž M., Billik P., Cherkezova-Zheleva Z., Criado J.M., Delogu F., Dutková E., Gaffet E., Gotor F.J. (2013). Hallmarks of mechanochemistry: From nanoparticles to technology. Chem. Soc. Rev..

[2] Ardila-Fierro K.J., Hernández J.G. (2021). Sustainability Assessment of Mechanochemistry by Using the Twelve Principles of Green Chemistry. Chem. Sus. Chem..

[3] Di Nardo T., Moores A. (2019). Mechanochemical amorphization of chitin: Impact of apparatus material on performance and contamination. Beilstein J. Org. Chem..

[4] Pérez-Merchán A.M., Rodríguez-Carballo G., Torres-Olea B., García-Sancho C., Maireles-Torres P.J., Mérida-Robles J., Moreno-Tost R. (2022). Recent Advances in Mechanochemical Pretreatment of Lignocellulosic Biomass. Energies.

[5] Bychkov A.L., Podgorbunskikh E.M., Bychkova E.S., Lomovsky O.I. (2019). Current achievements in the mechanically pretreated conversion of plant biomass. Biotechnol. Bioeng..

[6] Kuga S., Wu M. (2019). Mechanochemistry of cellulose. Cellulose.

[7] Barbash V.A., Yaschenko O.V., Alushkin S.V., Kondratyuk A.S., Posudievsky O.Y., Koshenko V.G. (2016). The Effect of Mechanochemical Treatment of the Cellulose on Characteristics of Nanocellulose Films. Nanoscale Res. Lett..

[8] Andrade I.H.P., Otoni C.G., Amorim T.S., Camilloto G.P., Cruz R.S. (2020). Ultrasound-assisted extraction of starch nanoparticles from breadfruit (*Artocarpus altilis* (Parkinson) Fosberg). Colloids Surf. A Physicochem. Eng..

[9] Anusha J.R., Fleming A.T., Valan Arasu M., Chul Kim B., Abdullah Al-Dhabi N., Yu K.-H., Justin Raj C. (2016). Mechanochemical synthesis of chitosan submicron particles from the gladius of *Todarodes pacificus*. J. Adv. Res..

[10] Akopova T.A., Popyrina T.N., Demina T.S. (2022). Mechanochemical Transformations of Polysaccharides: A Systematic Review. Int. J. Mol. Sci..

[11] Chen X., Yang H., Zhong Z., Yan N. (2017). Base-catalysed, one-step mechanochemical conversion of chitin and shrimp shells into low molecular weight chitosan. Green Chem..

[12] Di Nardo T., Hadad C., Van Nhien A.N., Moores A. (2019). Synthesis of high molecular weight chitosan from chitin by mechanochemistry and aging. Green Chem..

[13] Hou D.-F., Li M.-L., Li P.-Y., Zhou L., Zhang K., Liu Z.-Y., Yang W., Yang M.-B. (2023). Efficient Conversion of Cellulose to Thermoplastics by Mechanochemical Esterification. ACS Sustain. Chem. Eng..

[14] Vakili M., Qiu W., Cagnetta G., Huang J., Yu G. (2021). Mechanochemically oxidized chitosan-based adsorbents with outstanding Penicillin G adsorption capacity. J. Environ. Chem. Eng..

[15] Zhao H., Kwak J.H., Wang Y., Franz J.A., White J.M., Holladay J.E. (2006). Effects of Crystallinity on Dilute Acid Hydrolysis of Cellulose by Cellulose Ball-Milling Study. Energy Fuels.

[16] Hall M., Bansal P., Lee J.H., Realff M.J., Bommarius A.S. (2010). Cellulose crystallinity—A key predictor of the enzymatic hydrolysis rate. FEBS J..

[17] Lemos P.V.F., Barbosa L.S., Ramos I.G., Coelho R.E., Druzian J.I. (2018). The important role of crystallinity and amylose ratio in thermal stability of starches. J. Therm. Anal. Calorim..

[18] Zhu X., He Q., Hu Y., Huang R., Shao N. (2018). A comparative study of structure, thermal degradation, and combustion behavior of starch from different plant sources. J. Therm. Anal. Calorim..

[19] Li Y., Yan C., Chen Y., Han X., Shao Z., Qi H., Li H., Nishiyama Y., Hu T., Chen P. (2023). The major role of London dispersion interaction in the assembly of cellulose, chitin, and chitosan. Cellulose.

[20] O’Sullivan A.C. (1997). Cellulose: The structure slowly unravels. Cellulose.

[21] Emenike E.C., Iwuozor K.O., Saliu O.D., Ramontja J., Adeniyi A.G. (2023). Advances in the extraction, classification, modification, emerging and advanced applications of crystalline cellulose: A review. Carbohydr. Polym. Technol. Appl..

[22] Hermans P.H., Weidinger A. (1946). On the recrystallization of amorphous cellulose. J. Am. Chem. Soc..

[23] Ago M., Endo T., Hirotsu T. (2004). Crystalline transformation of native cellulose from cellulose I to cellulose II polymorph by a ball-milling method with a specific amount of water. Cellulose.

[24] Ago M., Endo T., Okajima K. (2007). Effect of solvent on morphological and structural change of cellulose under ball-milling. Polym. J..

[25] Kocherbitov V., Ulvenlund S., Kober M., Jarring K., Arnebrant T. (2008). Hydration of microcrystalline cellulose and milled cellulose studied by sorption calorimetry. J. Phys. Chem. B..

[26] Klemm D., Schmauder H.P., Heinze T., DeBaets S., Vandamme E.J., Steinbüchel A. (2002). Cellulose. Biopolymers. Polysaccharides II: Polysaccharides from Eukaryotes.

[27] Rosenau T., Potthast A., Sixta H., Kosma P. (2001). The chemistry of side reactions and byproduct formation in the system NMMO/cellulose (Lyocell process). Prog. Polym. Sci..

[28] Ahn Y., Song Y., Kwak S.Y., Kim H. (2016). Highly ordered cellulose II crystalline regenerated from cellulose hydrolyzed by 1-butyl-3-methylimidazolium chloride. Carbohydr. Polym..

[29] Holland S., Tuck C., Foster T. (2018). Selective recrystallization of cellulose composite powders and microstructure creation through 3D binder jetting. Carbohydr. Polym..

[30] Pita-Vilar M., Concheiro A., Alvarez-Lorenzo C., Diaz-Gomez L. (2023). Recent advances in 3D printed cellulose-based wound dressings: A review on in vitro and in vivo achievements. Carbohydr. Polym..

[31] Qiao Y., Zhai C., Liu F., Chen L., Na H., Chen J., Zhu J. (2020). Highly efficient microwave driven assisted hydrolysis of cellulose to sugar with the utilization of ZrO2 to inhibit recrystallization of cellulose. Carbohydr. Polym..

[32] Tsurkan M.V., Voronkina A., Khunyk Y., Wysokowski M., Petrenko I., Ehrlich H. (2021). Progress in chitin analytics. Carbohydr. Polym..

[33] Fernando L.D., Dickwella Widanage M.C., Penfield J., Lipton A.S., Washton N., Latgé J.-P., Wang P., Zhang L., Wang T. (2021). Structural Polymorphism of Chitin and Chitosan in Fungal Cell Walls From Solid-State NMR and Principal Component Analysis. Front. Mol. Biosci..

[34] Noishiki Y., Takami H., Nishiyama Y., Wada M., Okada S., Kuga S. (2003). Alkali-induced conversion of β-chitin to α-chitin. Biomacromolecules.

[35] Faria R.R., Guerra R.F., de Sousa Neto L.R., Motta L.F., de Faria Franca E. (2016). Computational study of polymorphic structures of α- and β-chitin and chitosan in aqueous solution. J. Mol. Graph..

[36] Huang J., Zhong Y., Weia P., Cai J. (2021). Rapid dissolution of β-chitin and hierarchical self-assembly of chitin chains in aqueous KOH/urea solution. Green Chem..

[37] Zobel H.F. (1988). Starch crystal transformations and their industrial importance. Starch.

[38] Dome K., Podgorbunskikh E., Bychkov A., Lomovsky O. (2020). Changes in the crystallinity degree of starch having different types of crystal structure after mechanical pretreatment. Polymers.

[39] Robyt J.F., Fraser-Reid B., Tatsuta K., Thiem J. (2008). Starch: Structure, properties, chemistry, and enzymology. Glycoscience.

[40] Trinh K.S. (2015). Recrystallization of starches by hydrothermal treatment: Digestibility, structural, and physicochemical properties. J. Food Sci. Technol..

[41] Miao M., Jiang B., Zhang T. (2009). Effect of pullulanase debranching and recrystallization on structure and digestibility of waxy maize starch. Carbohydr. Polym..

[42] Pratiwi M., Nur Faridah D., Lioe H.N. (2017). Structural changes to starch after acid hydrolysis, debranching, autoclaving-cooling cycles, and heat moisture treatment (HMT): A review. Starch.

[43] Zhu J.Y., Wang G.S., Pan X.J., Gleisner R. (2009). Specific surface to evaluate the efficiencies of milling and pretreatment of wood for enzymatic saccharification. Chem. Eng. Sci..

[44] Tyufekchiev M., Kolodziejczak A., Duan P., Foston M., Schmidt-Rohrb K., Timko M.T. (2019). Reaction engineering implications of cellulose crystallinity and water-promoted recrystallization. Green Chem..

[45] Salem K.S., Kasera N.K., Rahman M.A., Jameel H., Habibi Y., Eichhorn S.J., French A.D., Pal L., Lucia L.A. (2023). Comparison and evaluation of methods for cellulose crystallinity determination. Chem. Soc. Rev..

[46] Cuong H.N., Minh N.C., Hoa N.V., Trung T.S. (2016). Preparation and characterization of high purity β-chitin from squid pens (*Loligo chenisis*). Int. J. Biol. Macromol..

[47] (2021). Particle Size Analysis Image Analysis Methods—Part 2: Dynamic Image Analysis Methods.

[48] Krumbein W.C., Sloss L.L. (1963). Stratigraphy and Sedimentation.

[49] Brunauer S., Emmett P.H., Teller E. (1938). Adsorption of gases in multimolecular layers. J. Am. Chem. Soc..

[50] Segal L., Creely J.J., Martin A.E., Conrad C.M. (1959). An empirical method for estimating the degree of crystallinity of native cellulose using the X-ray diffractometer. Text. Res. J..

[51] Nara S., Mori A., Komiya T. (1978). Study on Relative Crystallinity of Moist Potato Starch. Starch.

[52] Podgorbunskikh E.M., Dome K.V., Bukhtoyarov V., Bychkov A.L. (2022). X-ray Diffraction for Detecting Starch Adulteration and Measuring the Crystallinity Indices of the Polymorphic Modifications of Starch. Health Food Biotechnol..

[53] Focher B., Beltrame P.L., Naggi A., Torri G. (1990). Alkaline N-deacetylation of chitin enhanced by flash treatments. Reaction kinetics and structure modifications. Carbohydr. Polym..

[54] Podgorbunskikh E.M., Bychkov A.L., Bulina N.V., Lomovskii O.I. (2018). Disordering of the crystal structure of cellulose under mechanical activation. J. Struct. Chem..

[55] Podgorbunskikh E., Kuskov T., Matveeva A., Ulihin A., Bychkov A., Lomovsky I., Polienko Y. (2023). Disordering of Starch Films as a Factor Influencing the Release Rate of Biologically Active Substances. Polymers.

[56] French A.D. (2014). Idealized powder diffraction patterns for cellulose Polymorphs. Cellulose.

[57] Liang X.H., Gu L.Z., Ding E.Y. (1993). Recrystallization behavior of cellulose and lignocellulose from *Pinus massoniana*. Wood Sci. Technol..

[58] Rietveld H.M. (1969). A profile refinement method for nuclear and magnetic structures. J. Appl. Cryst..

